# GV1001 inhibits cell viability and induces apoptosis in castration-resistant prostate cancer cells through the AKT/NF-κB/VEGF pathway

**DOI:** 10.7150/jca.34859

**Published:** 2019-10-17

**Authors:** Yong Hyun Park, Ae Ryang Jung, Ga Eun Kim, Mee Young Kim, Jae Woo Sung, Dongho Shin, Hyuk Jin Cho, U-Syn Ha, Sung-Hoo Hong, Sae Woong Kim, Ji Youl Lee

**Affiliations:** 1 Department of Urology, Seoul St. Mary's Hospital, College of Medicine, The Catholic University of Korea; 2 Cancer Research Institute, College of Medicine, The Catholic University of Korea

**Keywords:** castration-resistant prostate cancer, GV1001, AKT/NF-κB/VEGF signaling pathway

## Abstract

**Purpose:** We examined the effect of GV1001 in castration castration-resistant prostate cancer (CRPC) cell growth and invasion and explored the potential molecular mechanisms of action.

**Materials and Methods:** The *in vitro* anti-cancer effects of GV1001 in CRPC cells were examined using cell viability assay, TUNEL assay, and flow cytometry analysis. To evaluate the effects of GV1001 on different steps of angiogenesis, wound healing assay, transwell invasion assay, endothelial cell tube formation assay, and western blot analysis were performed. Finally, the anti-cancer effects of GV1001 on tumor growth *in vivo* were examined in a CRPC xenograft model.

**Results:** GV1001 inhibited cell viability and induced apoptosis in CRPC cells *in vitro,* accompanied by down-regulation of Bcl-2 and caspase-3. GV1001 also inhibited different steps of angiogenesis, such as migration, invasion, and endothelial tube formation, along with decreased expression of MMP-2, MMP-9, and CD31 and increased expression of TIMP-1 and TIMP-2. Mechanistically, GV1001 significantly decreased the levels of phosphorylated AKT, phosphorylated p65, and VEGF in CRPC cells in a dose-dependent manner. GV1001 was effective in suppressing tumor growth and inducing apoptosis in a CRPC xenograft mouse model.

**Conclusions:** Our data demonstrated that GV1001 inhibited cell viability, induced apoptosis, and inhibited angiogenesis in CRPC cells by inhibition of the AKT/NF-κB/VEGF signaling pathway.

## Introduction

Prostate cancer is the most common cancer in men in the United States, and the incidence of prostate cancer has been rapidly increasing over the last few years in Korea 1, 2. Androgen deprivation therapy is the standard of care for advanced or metastatic disease due to its good clinical response, however, this good response only lasts on average 2-3 years, with eventual progression toward castration-resistant prostate cancer (CRPC) 3, 4. Since the publication of two pivotal trials, docetaxel-based chemotherapy has been the standard treatment for patients with CRPC 5, 6. In addition to docetaxel-based chemotherapy, several new agents with different mechanisms of action have shown favorable clinical outcomes compared with standard of care or placebo in randomized controlled trials of CRPC. These agents include the new taxane, cabazitaxel 7; second-generation hormonal agents, abiraterone acetate 8, 9 and enzalutamide 10; the immunotherapeutic agent, sipuleucel-T 11; and the bone-targeting agent, radium-223 12. Despite these improvements in the last 5 years, additional treatment options are still needed to prolong the survival of patients with CRPC, especially those with resistance to these existing treatments.

GV1001 consists of small 16-mer peptides that is derived from the human telomerase enzyme (hTERT 611-626; EARPALLTSRLRFIPK), and is undergoing clinical trials as a cancer vaccine 13. GV1001 has been shown to induce specific immune response in patients with pancreatic cancer, non-small cell lung cancer, melanoma, and hepatocellular carcinoma in several phase I/II clinical trials. Although all of these clinical trials have examined the anticancer effect of GV1001 as a peptide vaccine, unexpected intracellular localization of GV1001 was observed. Through its interaction with HSP70 and HSP90, GV1001 can penetrate the cell membrane and localize in the cytoplasm. These findings suggest a possible role of GV1001 as a cell-penetrating peptide 14.

Given the demonstrated ability of GV1001 to penetrate cancer cells and directly interact with important signaling molecules, we speculated that GV1001 might have anti-cancer activity in cancer cells, including prostate cancer cells. In this study, we examined the effects of GV1001 on CRPC cell growth and invasion and explored the potential molecular mechanisms of action. Furthermore, the *in vivo* efficacy of GV1001 was investigated using a xenograft mouse model.

## Materials and Methods

### Cells and reagents

Human CRPC cell lines, DU145 and PC3, were purchased from the Korean Cell Line Bank (Seoul, Korea) and cultured in Dulbecco's Modified Eagle's Medium (DMEM) containing 10% fetal bovine serum (FBS), and penicillin (100 U/ml) at 37°C in a humidified 5% CO_2_ incubator. Human umbilical vein endothelial cells (HUVECs) were purchased from Life Technologies (Carlsbad, CA, USA) and cultured in Medium 200 (Invitrogen, Carlsbad, CA, USA) containing the LVES-supplement (Invitrogen). GV1001 (molecular weight, 1,686 g/mol) was supplied as a freeze-dried peptide produced under good manufacturing practice conditions by GemVax & Kael (Seongnam, Korea). GV1001 was stored at -20°C and thawed in phosphate buffer solution (maximum solubility: ~100 mg/mL in saline at ambient conditions).

### Cell viability assay

DU145 (4 x 10³/well) and PC3 cells (5 x 10³/well) were seeded in 96-well plates. After 24 h of incubation, cells were treated with GV1001 (0, 50, 100, 150, or 200 µM), and plates were incubated for 48 h at 37°C. The wells were washed once with PBS, and then 90 µL of culture medium was added to each well. Next, 10 µl PrestoBlue™ Cell Viability Reagent (Invitrogen) was added to each well, and the plate was incubated for 2 h at 37°C. The optical density (OD) was measured with an enzyme-linked immunosorbent assay (ELISA) plate reader and OD value of 570 nm to 600 nm was calculated. Each experiment was performed in three wells and repeated at least three times.

### TUNEL assay

DU145 (3 x 10⁴/well) and PC3 cells (4 x 10⁴/well) were seeded into a Nunc Lab-Tak chamber slide system (Thermo Scientific, Rockford, IL, USA). After 24 h of incubation, cells were treated with GV1001 (0, 100, or 200 µM) for 48 h. Terminal deoxynucleotidyl transferase-mediated deoxyuridine triphosphate nick end labeling (TUNEL) (Roche Diagnostics, Mannheim, Germany) was used to identify apoptotic cell death. Cells were fixed with 4% paraformaldehyde for 1 h at room temperature. After washing with PBS, cells were permeabilized in PBS containing 0.1% Triton X-100 and 0.1% sodium citrate for 5 min on ice and incubated with a mixture of TdT solution and fluorescein isothiocyanate dUTP solution in a humidified chamber for 1 h at 37°C. Cells treated with DNase served as positive controls. The samples were stained with 4′, 6-diamidino-2-phenylindole (DAPI; Vector, Peterborough, England) to visualize cell nuclei, and stained cells were examined using an Olympus BX51 microscope (Olympus Optical Co. Ltd., Tokyo, Japan). Ten different areas were randomly selected; the numbers of TUNEL-positive cells were counted, and the ratio of TUNEL-positive cells to total cells was calculated.

### Flow cytometry analysis

DU145 and PC3 cells were treated with GV1001 (0, 100, or 200 µM) for 48 h. After harvesting, cells were resuspended in 500 µL 1X binding buffer and were stained with Annexin V (5 µL) and PI (10 µL) (BD Biosciences, San Jose, CA, USA) for 15 min at 4°C in the dark. The samples were analyzed immediately by flow cytometry (FACScanto II, Becton Dickinson, San Jose, CA, USA).

### Wound healing assay

Cells were seeded in a six-well plate at a density of 5 x 10^5^ cells/well in culture medium. After 24 h, the cell layer was scratched with a 200 µL pipette tip, and the plates were washed with PBS twice to remove detached cells. Fresh culture medium containing different concentrations of GV1001 (0, 100, or 200 µM) was added to wells. At 0, 24, and 48 h later, the wound area was photographed using an Olympus BX51 microscope (Olympus Optical Co. Ltd.).

### Transwell invasion assay

Chemotactic motility of cells was determined using transwell chambers (6.5 mm insert, 8.0 µm pore size; Corning, NY, USA). The upper chamber was coated with Matrigel (1:9, Matrigel:DMEM) and dried at room temperature for 2 h. DU145 and PC3 cells were then seeded into the upper chamber with serum-free-DMEM containing GV1001 (0, 100, or 200 µM). Basal medium containing 10% FBS was added to the lower chambers as a chemoattractant. After 48 h of incubation, non-migrating cells were removed with cotton swabs, and migrating cells were stained with 0.5% crystal violet solution (Sigma-Aldrich, St. Louis, MO, USA). Cells were dissolved in 20% acetic acid, and solubilization-produced color was measured with an ELISA plate reader at 570 nm.

### Endothelial tube formation assay

HUVECs (5 x 10⁴cells/well) were seeded into 48-well plates pre-coated with Matrigel in 150 µL EBM-2 medium (Lonza, Walkersville, MD, USA) containing low serum (5% FBS), bFGF (35 ng/mL) and VEGF (1 ng/mL). After 24 h, cells were treated with GV1001 (0, 100, or 200 µM) and incubated for 18 h at 37°C, and tube formation was evaluated.

### Western blot analysis

Treated cells were lysed in ice-cold RIPA buffer (Cell Signaling Technology, Danvers, MA, USA) containing ethylenediamine tetraacetic acid-free protease inhibitor cocktail (Roche Diagnostics). Equal amounts of samples (30 µg of protein) were separated on a NuPAGE 4-12% bis-tris gel (Invitrogen) and then transferred onto a nitrocellulose membrane. After blocking, membranes were incubated with primary antibodies, including rabbit anti-MMP2 (1:1,000, Santa Cruz Biotechnologies, Santa Cruz, CA, USA), mouse anti-MMP9 (1:1,000, Santa Cruz Biotechnologies), rabbit anti-TIMP1 (1:2,000, Abcam, Cambridge, MA, USA), mouse anti-TIMP2 (1:2,000, Abcam), rabbit anti-VEGF (1:1,000, Santa Cruz Biotechnologies), rabbit anti-CD31 (1:1,000, Abcam), and mouse anti-β-actin (1:1000, Abcam). After washing, the membrane was incubated with secondary antibody, and bands were visualized using ECL methods (Amersham, Arlington Height, IL, USA). Protein bands were detected using LAS-1000 (Fujifilm Medical Systems USA, Stamford, CT, USA).

### *In vivo* xenograft models of human CRPC

All animal experiments were approved by the Institutional Animal Care and Use Committee (IACUC-2015-0129-01) at The Catholic University of Korea. Six-week-old male BALB/c nude mice (n = 20, Orient Bio Co., Seongnam, Korea) were subcutaneously injected with DU145 (4 x 10^6^ cells) and PC3 (5 x 10^6^ cells) into mouse flanks (10 mice per cell line). When the tumors reached 10 mm in diameter, subcutaneous injection with 100 µL PBS or 1 mg/kg GV1001 in 100 µL PBS (5 mice per treatment group) was performed daily for 4 weeks. Tumor sizes were measured three times a week, and tumor volume was calculated according to the following formula: volume (mm³) = (length x width²)/2.

### Immunohistochemistry

Tumor tissues were fixed in 4 % paraformaldehyde for 24 h at 4°C, and tissue paraffin sections (5 µm) were incubated overnight. Slides were immunostained with primary antibodies against vascular endothelial growth factor (VEGF; 1:200, Santa Cruz Biotechnologies), CD31 (1:200, Abcam), and DAPI (Vector Labs, Burlingame, CA, USA). Fluorescent imaging was performed with an Olympus BX51 fluorescence microscope (magnification of x200), and images were analyzed using ZEN 2009 software (Carl Zeiss, Jena, Germany).

### Statistical analysis

Statistical analyses were performed using SPSS® version 24.0 software (IBM, Armonk, NY, USA). All experiments were repeated at least three times, and data are expressed as mean ± standard deviation (SD). Statistical significance was determined by independent T-test, and P-values ≤0.05 were considered statistically significant.

## Results

### GV1001 inhibited cell viability and induced apoptosis in CRPC cells

**Figure 1:**
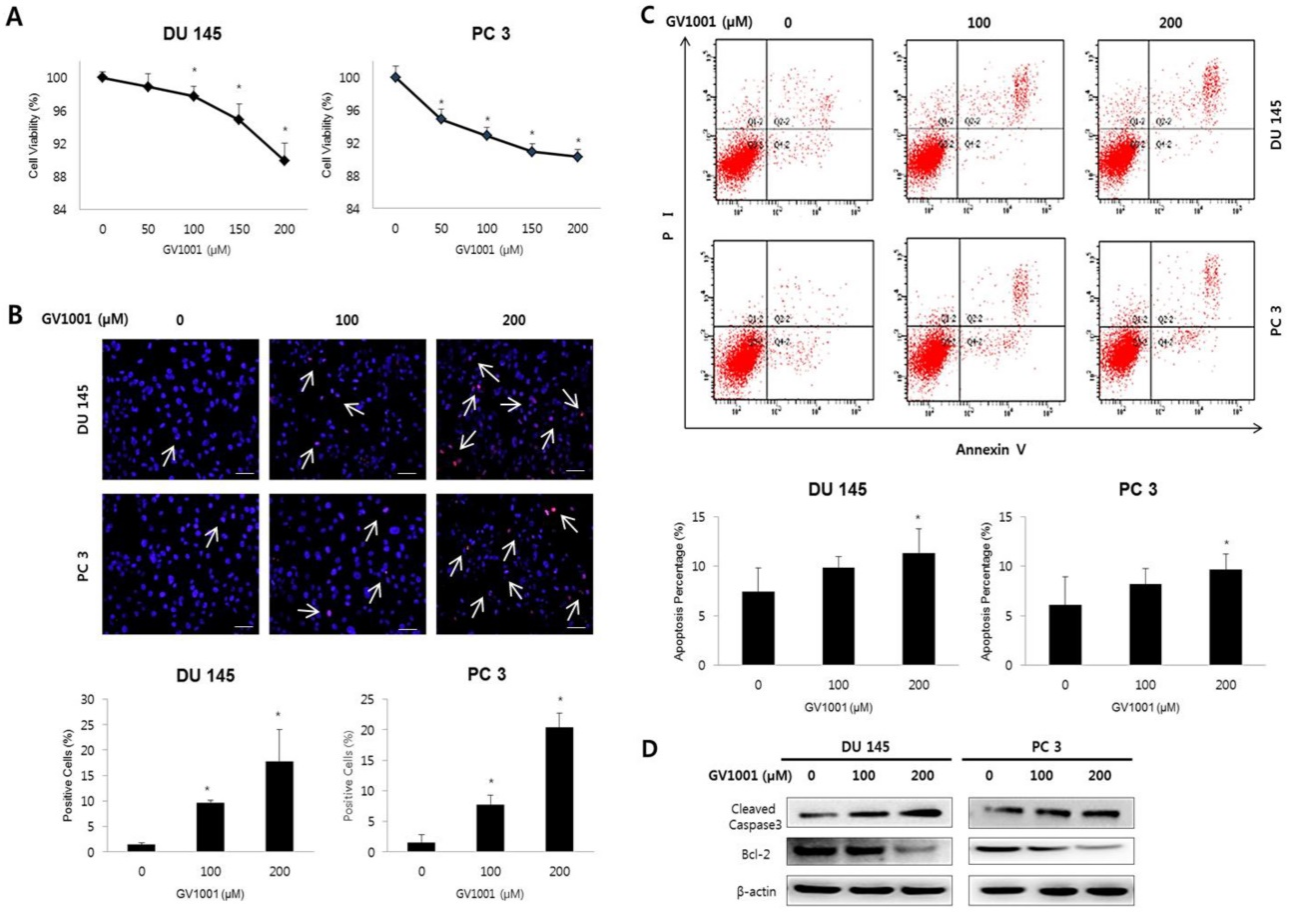
** GV1001 inhibits cell viability and induces apoptosis in CRPC cells.** (A) Human CRPC cell lines were treated with GV1001 (0, 50, 100, 150, and 200 µM) for 48 h and cell viability was assessed. (B) Apoptotic cells were evaluated by TUNEL assay in DU145 and PC3 cells treated as indicated. The ratio of TUNEL positive cells was calculated as the number of cells with positive TUNEL staining (red) divided by the number of cells with positive cells staining (blue). All images are 400x magnification. (C) Evaluation of GV1001-induced apoptosis in CRPC cells by flow cytometry. The percentages of apoptotic cells were analyzed by dot plot. All data were expressed as mean ± standard deviation. *P<0.05 compared with controls. (D) Western blot analysis for Bcl-2 and cleaved caspase-3 in CRPC cells after GV1001 treatment. The β-actin housekeeping protein was used as an internal control.

We first examined the effect of GV1001 on cell viability of CRPC cells using PrestoBlue™ Cell Viability Reagent. As shown in Fig. 1A, treatment with GV1001 induced a dose-dependent reduction of cell viability in both DU145 and PC3 cells (P<0.05).

To determine whether the reduction of cell viability induced by GV1001 was associated with apoptosis, we performed TUNEL assay and Annexin V-FITC/PI staining performed in CRPC cells treated with various concentrations of GV1001 (0, 100, and 200 µM). Figure 1B shows representative images for each treatment group; apoptotic cells were stained red, and cell nuclei were stained blue. The TUNEL-positive DU145 cells in the 0, 100, and 200 µM samples represented 1±0.35, 10±0.44, and 18±6.23% of the total cells, respectively. Similarly, the TUNEL-positive PC3 cells treated with 0, 100, and 200 µM GV1001 comprised 2±1.24, 8±1.46, and 20±2.32% of the total, respectively (Fig. 1C). TUNEL-positive cells were significantly increased in response to GV1001 in a dose-dependent manner in both DU145 and PC-3 cells (P<0.05). Furthermore, as shown in Fig. 1C, the proportions of early and late apoptotic DU145 cells treated with 0, 100 and 200 0 µM GV1001 were 7.44±2.41, 9.89±1.08, and 11±2.42%, respectively (P<0.05). Similarly, the proportions of early and late apoptotic cells in PC3 cells treated with 0, 100 and 200 0 µM GV1001 were 6.10±2.86, 8.24±1.56, and 9.66±1.56%, respectively (P<0.05). Together, these results indicated that GV1001 significantly decreased cell viability and induced apoptosis in CRPC cells in a dose-dependent manner.

We next examined the effects of GV1001 on apoptosis-related proteins Bcl-2 and cleaved caspase-3 using western blot (Fig. 1D). GV1001 induced a dose-dependent increase in the expression level of cleaved caspase-3 protein and led to a dose-dependent decrease in the expression level of Bcl-2 protein.

### GV1001 inhibited invasion and migration capacity of CRPC cells

**Figure 2:**
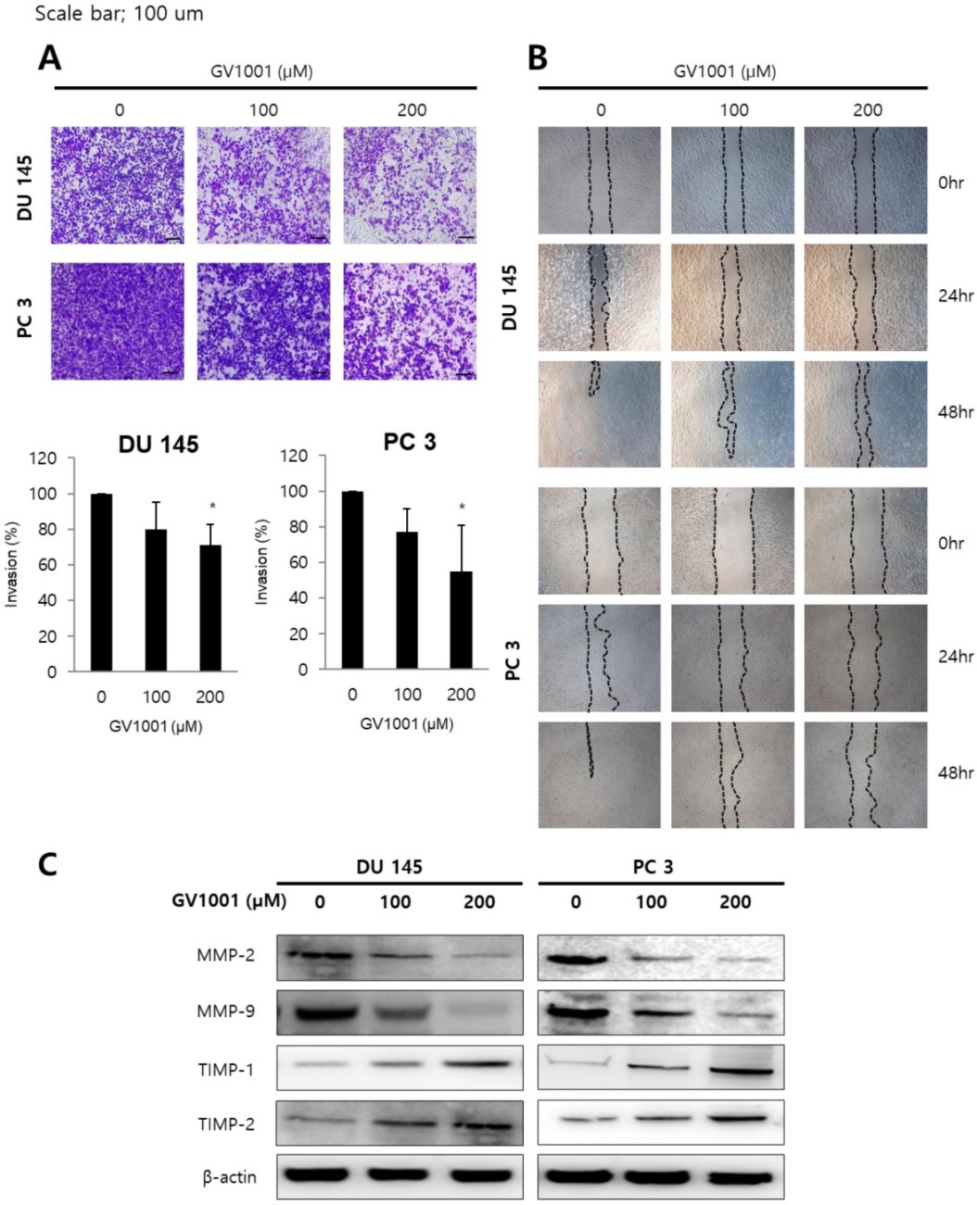
** GV1001 inhibits invasion and migration capacity of CRPC cells.** (A) Representative microscopic images of cells that had migrated into the lower chamber are shown at 200x magnification. The migrated cells were quantified using a bar graph. Data are expressed as mean ± standard deviation. *P<0.05 compared with controls. (B) At 0, 24, and 48 h after scratching in wound healing assays, cell migration was assessed by microscopy (100x magnification). (C) Western blot analysis for MMP-2, MMP-9, TIMP-1 and TIMP-2 in CRPC cells after GV1001 treatment. The β-actin housekeeping protein was used as an internal control.

Since invasion and migration capacity are closely associated with metastatic potential, we next assessed the effects of GV1001 on invasion and migration of CRPC cells using transwell invasion assay and wound healing assay. As shown in Fig. 2A, GV1001 treatment (0, 100, and 200 µM) significantly inhibited the invasion of DU145 (100±0%, 79.79±15.49%, and 71.23±11.30%) and PC3 cells (100±0%, 77.12±13.15%, and 54.62±26.07%) in a dose-dependent manner. In addition, the migration capacity of DU145 and PC3 cells was reduced in a dose- and time-dependent manner compared with control group (Fig. 2B). To confirm the above results, western blot analysis was performed for expression of MMP and TIMP proteins, which are important in cell invasion and migration. As shown in Fig. 2C, GV1001 significantly decreased MMP-2 and MMP-9 expression in CRPC cells, and increased TIMP-1 and TIMP-2 expression.

### GV1001 inhibited angiogenesis

**Figure 3:**
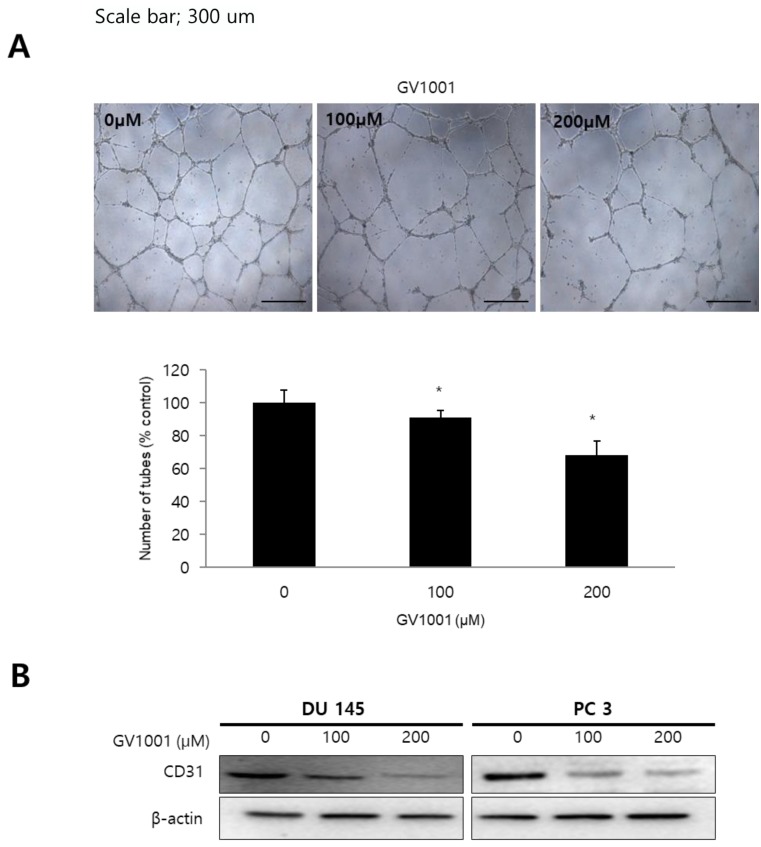
** GV1001 inhibits angiogenesis.** (A) The endothelial tube formation assay was performed in CRPC cells treated with various concentrations of GV1001 (0, 100, and 200 µM). Representative microscopic images of changes in cell morphology are shown at 50x magnification. The number of tube-like structures was counted, and tube formation was calculated as a percentage of the control. Data are expressed as mean ± standard deviation. *P<0.05 compared with controls. (B) Western blot analysis for CD31 protein in CRPC cells treated with GV1001. The β-actin was used as a loading control.

To determine the effect of GV1001 on angiogenesis *in vitro*, we performed tube formation assays in HUVECs. Treatment of HUVECs with GV1001 (0, 100, and 200 µM) decreased the number of capillary-like tube structures and resulted in broken and shortened tubes compared with the control group (Fig. 3A). We also evaluated the protein expression of CD31, an angiogenic marker, in CRPC cells treated with different concentrations of GV1001. As shown in Fig. 3B, GV1001 treatment downregulated the expression level of CD31 in a dose-dependent manner. Together, these findings confirmed that GV1001 inhibited different steps of angiogenesis, including migration, invasion, and tube formation *in vitro*.

### GV1001 exhibits anticancer effects in CRPC cells through the AKT/NF-κB/VEGF pathway

**Figure 4:**
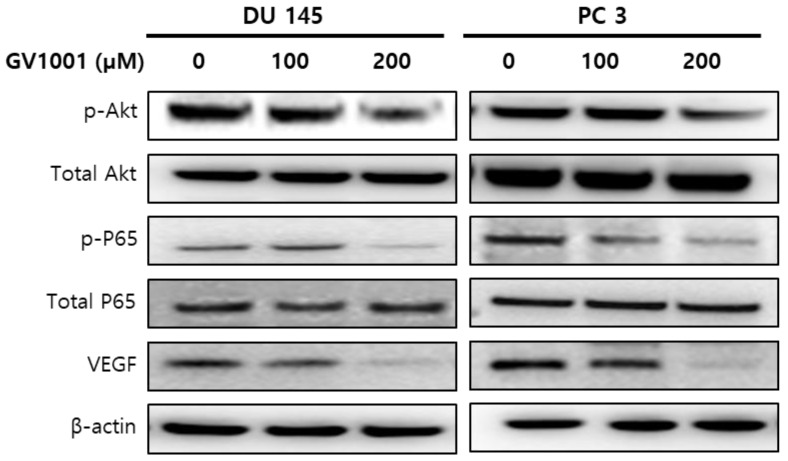
GV1001 exhibits anticancer effects in CRPC cells through the AKT/NF-κB/VEGF pathway.

Since the phosphatidylinositol 3-kinase (PI3K)/AKT survival pathway plays an important role in carcinogenesis and angiogenesis 15, we performed western blot analysis to evaluate the expression level of AKT in CRPC cells treated with GV1001. As shown in Fig. 4, GV1001 treatment resulted in dose-dependent inactivation of AKT in CRPC cells. NF-κB is an important downstream effector of the PI3K/AKT survival pathway 16, and we next examined whether NF-κB and VEGF were affected by GV1001 treatment. The results showed that the levels of phosphorylated p65 and VEGF were significantly decreased in CRPC cells treated with GV1001.

### GV1001 exhibits anticancer effects in CRPC xenograft model

**Figure 5:**
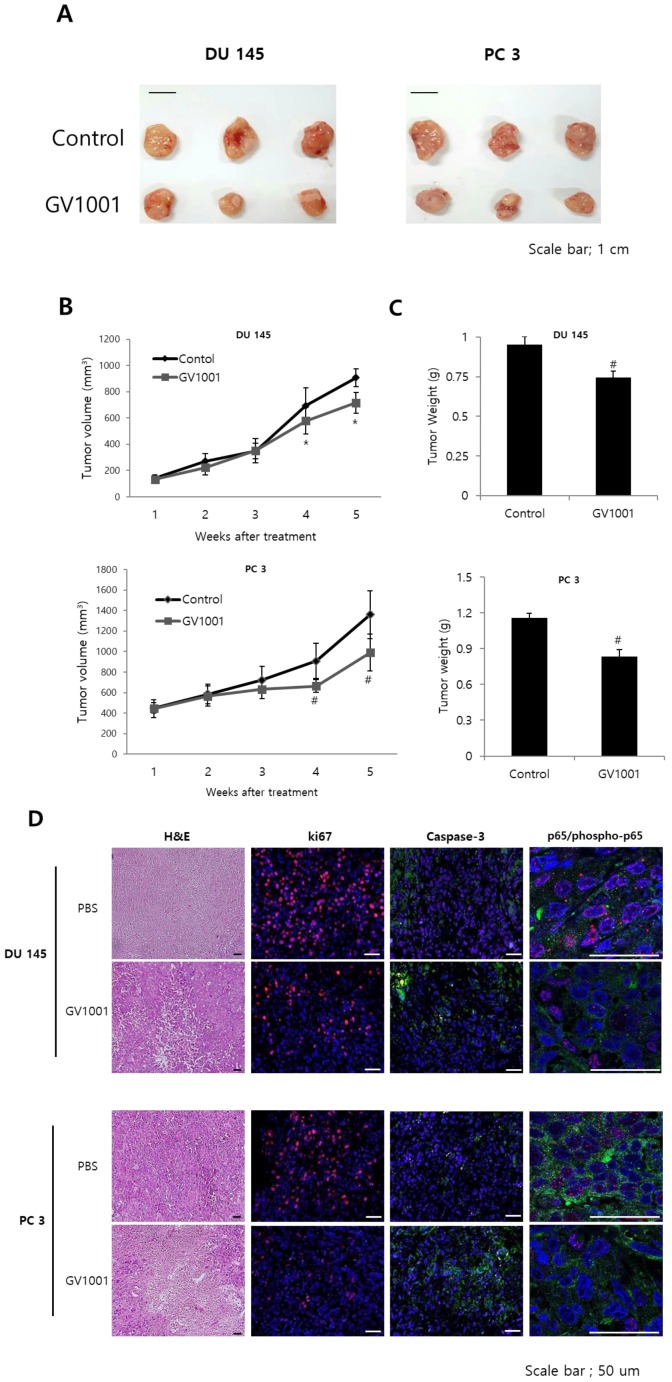
** GV1001 exhibits anticancer effects in a CRPC xenograft model.** (A) Representative images of tumors from the DU145 and PC3 xenograft mouse model. (B, C) Mean tumor volume and weight are shown. (D) Tumors were excised and processed for hematoxylin and eosin staining and immunohistochemical staining for Ki-67, CD31, and p65. Representative images are shown at 400x.

To confirm the above findings demonstrating the anticancer activities of GV1001 *in vitro*, we next examined the *in vivo* anticancer efficacy of GV1001 in a DU145 and PC3 xenograft mouse model. After 4 weeks of treatment, GV1001 significantly reduced the volume of tumors derived from both DU145 and PC3 cells compared with controls (Figs. 5A and B). The tumor weight in the GV1001 treatment group was lower than that in the control group (Fig. 5C). These findings indicated that GV1001 was effective in suppressing tumor growth in the CRPC xenograft model. We also evaluated proliferation and apoptosis-related protein expression in tumor sections from the xenograft mice and found that GV1001-treated tumors showed significantly decreased Ki-67 expression and increased cleaved caspase-3 expression compared with controls (Fig. 5D). In addition, phosphorylated p65 expression was significantly decreased in GV1001-treated tumors.

## Discussion

GV1001 was initially developed as a telomerase-specific peptide vaccine for pancreas and lung cancer 17, 18. Interestingly, Lee at al. found that GV1001 had an unexpected function as a cell-penetrating peptide, and the delivered GV1001 was predominantly located in the cytoplasm of the cells through its interaction with HSP70 and HSP90 14. Several researchers later reported anti-cancer effects of GV1001 in various cancer cells 14, 19, 20. In the present study, we demonstrated that GV1001 had potent effects in inhibition of cell viability, induction of apoptosis, and inhibition of angiogenesis in CRPC cells in a dose-dependent manner. Our *in vivo* study also demonstrated that GV1001 exhibited inhibitory effects on tumor growth in CRPC xenograft-bearing mice. The anti-cancer effects of GV1001 in CRPC cells may be mediated through inhibition of the AKT/NF-κB/VEGF pathway. To the best of our knowledge, this is the first report of the anticancer effects and biological mechanism of GV1001 in human CRPC cells.

Angiogenesis is an important step of tumorigenesis 21. Thus, inhibition of angiogenesis has become an effective treatment strategy for solid cancers. VEGF is a key regulator of endothelial cell proliferation, migration, invasion, vascular permeability, and vasodilation 22. In this study, we examined the role of GV1001 in tumor angiogenesis and found that GV1001 strongly inhibited tube formation. GV1001 also inhibited cell invasion and migration capacity and resulted in dose-dependent suppression of VEGF. To examine the underlying mechanisms of the anti-angiogenic effects of GV1001, we examined AKT and NF-κB protein expression. GV1001 markedly inhibited the expression of AKT and NF-κB in CRPC cells.

AKT is a serine/threonine kinase that regulates tumor cell proliferation, survival, bio-energetics, and angiogenesis by phosphorylation of its molecular targets, including factors in the NF-κB pathway 23. Inflammatory stimuli can activate the PI3K/Akt pathway in various cell types, leading to phosphorylation of IκBα. Phosphorylation and nuclear translocation of p65 lead to activation of NF-κB and production of VEGF 24. This is an important mechanism of pathologic angiogenesis, and GV1001 may inhibit the AKT/NF-κB/VEGF pathway and resulted in decreased tumor angiogenesis.

The precise mechanisms underlying the anti-cancer effects of GV1001 have not been completely elucidated. In addition to our data demonstrating effects on the AKT/NF-κB/VEGF pathway, GV1001 exhibits several potential biological mechanisms of action, with involvement in the TGF-β signaling pathway 25, the hypoxia-induced HIF-1α-VEGF signaling axis 19, and the ERK and p38 MAP kinase pathway 26. In addition, GV1001 inhibits gonadotropin-releasing hormone (GnRH) and 5α‑reductase, which are important in prostate cancer 27, 28. Kim et al. reported that GV1001 can be a therapeutic agent for testosterone-induced benign prostatic hyperplasia (BPH) 27. After establishing BPH in castrated rats via daily subcutaneous injections of testosterone propionate, GV1001 was administered for 4 weeks. The authors found that GV1001 significantly decreased prostate weight and inhibited 5α‑reductase activity in a dose-dependent manner. GV1001 is a ligand for GnRH receptor to selectively stimulate the Gαs/cAMP pathway and antagonize Gαq-coupled Ca^2+^ release by leuprolide acetate in prostate cancer 28. GV1001 might also exhibit anti-cancer effects in CRPC cells through these biological mechanisms.

In conclusions, we demonstrated that GV1001 inhibited cell viability, induced apoptosis, and inhibited angiogenesis in CRPC cells by inhibition of the AKT/NF-κB/VEGF signaling pathway. These results provide experimental evidence for GV1001 as a potential therapeutic agent of CRPC.
